# Comprehending the Health Informatics Spectrum: Grappling with System Entropy and Advancing Quality Clinical Research

**DOI:** 10.3389/fpubh.2017.00224

**Published:** 2017-09-14

**Authors:** Matthew I. Bellgard, Nigel Chartres, Gerald F. Watts, Steve Wilton, Sue Fletcher, Adam Hunter, Tom Snelling

**Affiliations:** ^1^Centre for Comparative Genomics, Murdoch University, Murdoch, WA, Australia; ^2^Health Informatics Society of Australia, North Melbourne, VIC, Australia; ^3^School of Medicine, University of Western Australia, Perth, WA, Australia; ^4^Lipid Disorders Clinic, Cardiometabolic Service, Royal Perth Hospital, Perth, WA, Australia; ^5^Centre for Neuromuscular and Neurological Disorders, University of Western Australia, Nedlands, WA, Australia; ^6^Perron Institute for Neurological and Translational Science, Nedlands, WA, Australia; ^7^Princess Margaret Hospital for Children, Perth, WA, Australia; ^8^Wesfarmers Centre of Vaccines and Infectious Diseases, Telethon Kids Institute, University of Western Australia, Perth, WA, Australia; ^9^Menzies School of Health Research and Charles Darwin University, Darwin, NT, Australia

**Keywords:** health, informatics, clinical research, information communication technology, clinical practice

Clinical research is complex. The knowledge base is information and data rich where value and success depend upon focused, well designed connectivity of systems achieved through stakeholder collaboration. Quality data, information, and knowledge must be utilized in an effective, efficient, and timely manner to affect important clinical decisions and communicate health prevention strategies. In recent decades, it has become apparent that information communication technology (ICT) solutions potentially offer multidimensional opportunities for transforming health care and clinical research. However, it is also recognized that successful utilization of ICT in improving patient care and health outcomes depends on a number of factors such as the effective integration of diverse sources of health data; how and by whom quality data are captured; reproducible methods on how data are interrogated and reanalyzed; robust policies and procedures for data privacy, security and access; usable consumer and clinical user interfaces; effective diverse stakeholder engagement; and navigating the numerous eclectic and non-interoperable legacy proprietary health ICT solutions in hospital and clinic environments (1, 2). This is broadly termed health informatics (HI).

We outline three scenarios from across the health spectrum where these issues are exemplified: (i) for a given clinical trial methodology and study design, the nature of how quality data is captured, by whom, how it is aggregated, reused and repurposed is just as critical as the data content itself. This becomes critical with the desire to simultaneously evaluate and optimize the effective and cost-effective use of new medications (3); (ii) in a systems biology context, clever strategies to combine disparate datasets at the gene, gene expression, protein as well as at a protein–protein interaction levels are essential to unlock underlying molecular mechanisms that affect routine clinical decisions (4); and (iii) in evidence-based medicine, encoding expert clinical knowledge into decision support systems and data standards for collecting diverse patient’s physiological measurements are critical to ensure effective cross jurisdictional data sharing for diseases (5).

These three examples highlight the potential broad spectrum of the role of ICT in health. Simply stated, at one end of the spectrum, health ICT systems are critical for the routine day-to-day running of hospitals and clinics. These systems are used by various health stakeholders for a diverse range of clinical services and administrative procedures. More recently, there is an increasing demand to reuse and repurpose health data contained within these ICT systems for clinical research and reporting such as compliance, efficiency metrics, funding of health programs, epidemiological studies, and health promotion. On the other end of the spectrum, clinical research embeds ICT and its application involving bioinformaticians, biostatisticians, and analytic workflow environments within research projects. There is a growing demand to embed outputs of this research as evidence to inform health-care policy and improve clinical practice.

The significant challenge is how we bridge these two ends of the spectrum. While the overall driver of improved patient outcomes is shared, the demands placed on available ICT systems for data capture, access, and analysis are usually beyond what they were originally designed for. We contend that the field of HI is the important bridge that delivers the promise spanning ICT spectrum in both health care and clinical research. We now explore the challenges in HI that need to be overcome.

## Key HI Challenges within the Current Environment

### Key Challenge 1: Defining HI

There are numerous broad definitions of HI. One such definition is that HI is “an evolving scientific discipline that deals with the collection, storage, retrieval, communication and optimal use of health and related data, information and knowledge” (6). The discipline draws on computational and information science methodologies and technologies to support clinical decision-making to improve health care. Such a broad definition has both advantages and disadvantages. On the one hand, this definition is a “catch all” for the spectrum of ICT in health care and clinical research. On the other, such a broad definition impacts a diverse range of health-related stakeholders from researchers, clinicians, nurses, public, allied health, health professionals, government departments, administrators, and software engineers. This presents a significant challenge of ensuring effective communication and uptake of robust HI.

### Key Challenge 2: Current Health ICT Ecosystems

In reality, health ICT ecosystems are largely fragmented (7, 8). For example, typically within a hospital ICT system environment, there are stand-alone systems, meaning that important health data are also siloed. Depending on the nature of these systems (some of which are as simple as spreadsheets), it is highly likely to contain significant data entry errors, duplications, inconsistencies, and incompleteness. The key challenge here is that fragmented ICT systems impedes the ability to monitor chronic diseases, effectively follow-up patients after hospital discharge, prevent avoidable complications (for example, hospital readmissions), or enable longitudinal epidemiological studies. This has a flow on cost burden effect and can inhibit efficiency gains within the health system. In Australia, numerous health-care business units (such as radiology, pharmacy, pathology, and radio oncology) typically have their own ICT systems that do not interface with each other, and most hospital systems do not interact with external systems, such as general practice clinics or private clinic rooms. Therefore, ownership and management of data become an important barrier between health-care business units and affect the quality of patient care. Furthermore, when proprietary systems are deployed and hosted by third parties, the ability of the client to exercise their ownership rights over their data requires clarification at the outset of the hosting arrangements.

### Key Challenge 3: Underlying Causes of Issues with Current ICT Ecosystems

Many papers and conferences addressing significant issues inherent in the challenges of introducing successful ICT ecosystems into the health sector continue to identify some key underlying causes for system failures and continuing difficulties in achieving meaningful connectivity within the health-care system, for example, see Ref. (9). These issues generally fall under the following 10 headings.

#### Leadership and Governance

Currently, the required degree of alignment of shared leadership and appropriate governance arrangements, across the many areas of responsibility, needed for systems synergies, are limited. Program management is equally important to project management to ensure shared learning of technical and interpersonal expertise.

#### Policy and Funding Models

Although health reform agendas mean to streamline policymaking and funding models, many stakeholders consider that very little has really been achieved that delivers any significant improvements into the way health systems operate. In this context, there are significant funding and resourcing pressures on any given state/national health system. The nature of these pressures unfortunately means that the focus reverts back to a business-as-usual paradigm within health systems. Furthermore, the current budgetary and operational pressures on the health sector restrict the ability of leadership within the sector to respond to contemporary challenges.

#### Regulatory Impediments

Existing and complex regulatory environments are viewed as a major issue where very little practical and beneficial change has been able to be introduced.

#### Productivity and Performance

It is recognized that significant progress has been made in reporting/compliance arrangements and systems that are focusing on transparency and accountability of health-care service providers. Given the current widespread lack of active use of data standards utilized within the fragmented health ICT ecosystem, it is difficult to harness the big data opportunities inherently available in health-care performance metrics (10). As such it is neither feasible nor practical to be able to use performance metrics to assess productivity in meaningful depth that could introduce transformative efficiencies into service delivery models.

#### Standards

Globally, there is much valuable work on developing open standards in health, for example, Ref. (11, 12). However, there remain many challenges in their widespread adoption related to limited funding, limited leadership capacity, widespread agreement, and limited workforce skills and resources. A particular issue concerns the focus on data collection and data entry rather than what we refer to as a more holistic approach to data management including the *purposeful application of collected data* to improve health outcomes.

#### Business Models and Processes—The Illusion of Risk Free Procurement

A significant barrier to the successful deployment of new systems is managing the transition from legacy ICT systems and data management processes in delivery of health services. This has further exacerbated the disparity between implemented ICT solutions and the business models and processes, which they purport to support. For instance, the procurement processes of health ICT solutions should be continually reviewed and iteratively refined along the dimensions of digital disruption, accountability, risk assessment, risk mitigation, risk averse strategies.

Evidence of potential suboptimal processes is highlighted by the patient journey through the health system, which invariably spans organizational and operational boundaries whose systems are typically not seamlessly connected to support the overall delivery of health care (9). In the case of rare disease diagnosis, a patient’s navigation through the health system is referred to more as an odyssey than a journey (3).

In addition, business model and process reform which is required systemically throughout the health system and much of which depends upon regulatory reform, is considered one of the most significant barriers to any beneficial transformation of the health system.

#### Sociotechnical Complexities

Sociotechnical complexities (complexities that span societal and technical boundaries) are inextricably linked to many aspects of business models and their associated business processes. Many of these complexities are inherently cultural in nature, in so far as many health workforce participants operate within long standing conventionally designed systems ecosystems. So while some progress continues to be achieved in specific situations, the big breakthroughs can only be achieved through large-scale business model and process reform as driven by regulatory change. If these are not addressed, then, for example, emerging trends such as patient empowerment *via* the measured self (13), the Internet of things (5), and personalized medicine will only see these complexities exacerbated (14).

Another key aspect of this concerns a real focus on business models, business processes, and systems, which collectively enable much more community engagement at all levels in consultation on matters such as prevention, patient care, diagnosis, treatment, management, privacy, and consent.

#### Infrastructure Component Connectivity

Technical and communication infrastructure is no longer viewed as the major issue as it was in recent times. It is clear that more effort needs to be made to connect existing infrastructure components to enable better communication between health-care service providers and so achieve more coordination of services.

#### Workforce

A barrier to success exists in the form of limited staff capacity across a range of administrative, clinical, research and technology disciplines to overcome the significant business-as-usual pressures of national health systems. This must be addressed to implement transformative change. ICT systems inherently can track performance, which can give rise to fear of inappropriate exposure for suboptimal clinical decision-making.

#### Clinical Research

There are limited virtual spaces where the health sector can interface with the research sector. Health departments do not have infrastructure to provide analytic environments for their big data, academic environments are typically not structured to handle health data, despite possessing the analytic capabilities.

## Case Study: Dementia

The Organisation for Economic Co-operation and Development (OECD) has recognized that there is clear potential to improve science and innovation systems through big data and open science for the prevention and care of dementia. In 2010, 35 million people worldwide were diagnosed with dementia with annual health costs estimated at USD 604 billion with the number of people diagnosed to exceed 115 million by 2050. The multifactorial nature of the condition requires the collection, storage, and processing of increasingly large and very heterogeneous datasets (behavioral, genetic, *-omics*, environmental, epigenetic, clinical data, brain imaging, and so forth) (10).

To successfully apply informatics systems to big data, current barriers, issues, and challenges need to be recognized and addressed along with implementing key critical success factors. For example, the OECD identified data sharing as the most significant barrier in managing dementia (15). The root cause of this significant barrier arises from current cultural, technical, administrative, regulatory, infrastructure, and financial obstacles that need to be overcome. In addition, data standards, data sharing, new analytic approaches, security and protecting privacy, along with approaches for engaging stakeholders and the public are critical factors for effectively and successfully harnessing big data. Hence, the future opportunity for big data in improving health-care systems requires carefully crafted strategies at both policy and ICT implementation levels across a broad range of HI challenges. In particular, regard needs to be paid to the established discipline of data governance, which is particularly important for providing a solid structural basis for managing human resources, processes, and technologies (1, 2).

## The Future Contribution of HI to Improving Health Outcomes

A learning health-care system requires a number of critical ingredients that can improve care of patients. These entail definition of clinical context, accurate collection of patient characteristics and outcome data, availability of decision support systems, utilization and application of real world data, and effective engagement of all stakeholders.

### Introducing a Guiding Model for the Role of HI to Span the Spectrum of ICT in Clinical Research

Owing to the current complexities and issues inherent in making substantial progress in improving health outcomes through the deployment of ICT enabling systems it is clear that there needs to be a better understanding of the role which HI plays.

**Figure 1:**
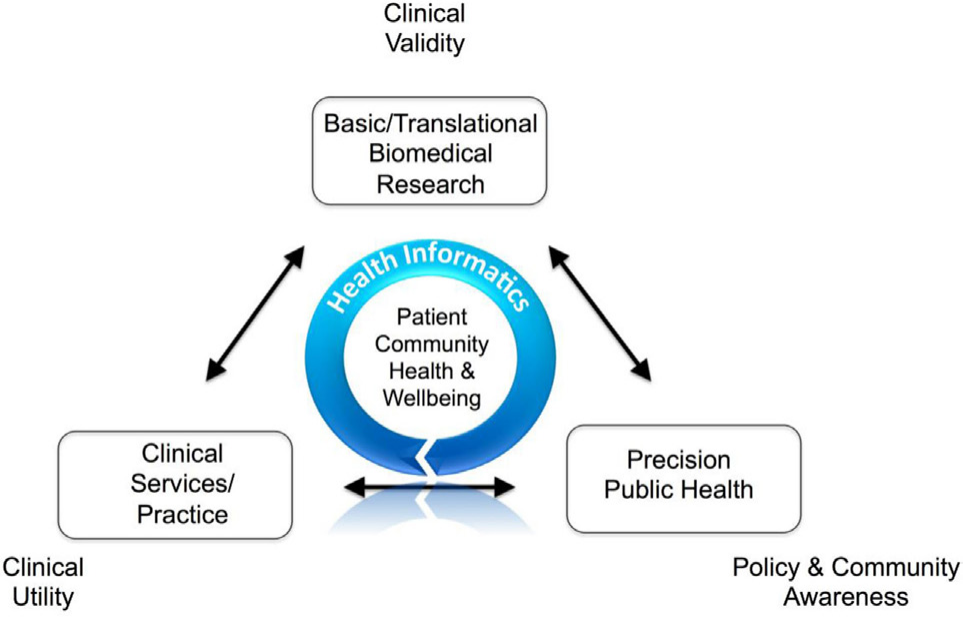
Health informatics role in clinical research.

Figure 1 provides an overview of a proposed guiding model highlighting the ideal role that HI plays in health care. Within this model, for example, clinical research will generate and analyze data such as a personalized genome sequence, to obtain clinical validity of candidate pathogenic mutations (16). The identified pathogenic mutation data are captured as one of myriad of patient phenotypes and patient reported outcomes to ascertain clinical utility, such as in a disease registry, e.g., Ref. (17, 18), as part of clinical services and practice, in a personalized medicine context (14). In the third axes, pathogenic mutations data can be aggregated in a de-identified manner across geographical locations to inform policy and community awareness (19) and undertaking important population health research.

## Conclusion and Future Perspective: Strategies for Shaping Effective and Sustainable Systems

From our experience, there are three key linked and iterative strategies for shaping and delivering successful systems. These are to:

- Facilitate a vision for shaping successful sustainable synergistic systems through shared leadership enabling collaborative stakeholder engagement;
- Recognize and address complexity through engaging stakeholders and the health workforce in identifying issues, problems, barriers, and potential solutions; and
- Create clever connected communities for the purposes of identifying and introducing innovative and informed investments in synergistic systems.

These, necessarily, need to be very skilfully planned, managed, and executed, which requires professional systems thinking HI practitioners who also have a very pragmatic working knowledge of the health system. This topic will be the subject of further commentary.

Information communication technology solutions must be discussed in an open and willing environment where risk is understood and carefully managed to facilitate strategic planning. These solutions must be designed to be able to apply open data standards and open system principles that promote interoperability, service oriented architectures, application programming interfaces, and appropriate assessment of legacy ICT systems (12, 20).

## Author Contributions

All the authors have contributed to this work.

## Conflict of Interest Statement

The authors declare that the research was conducted in the absence of any commercial or financial relationships that could be construed as a potential conflict of interest.
